# Feasibility of Post‐Operative Telehealth for Pediatric Surgical Patients in Malawi—A Mixed Methods Analysis

**DOI:** 10.1002/wjs.70448

**Published:** 2026-06-30

**Authors:** Madhushree Zope, Seraiah Mdala, Wiseman Phiri, Bvumi Tembo, Sella Mpata, Betty Yuggu, Bidali Nzira, Amarylis Mapurisa, Casey L. McAtee, Jed G. Nuchtern, Bip Nandi, Lily Gutnik

**Affiliations:** ^1^ Department of General Surgery University of Alabama at Birmingham General Surgery Birmingham Birmingham Alabama USA; ^2^ Kamuzu Central Hospital Pediatric Surgery Lilongwe Lilongwe Malawi; ^3^ Baylor College of Medicine Pediatric Hematology/Oncology Houston Houston Texas USA; ^4^ Baylor College of Medicine Pediatric Surgery Houston Houston Texas USA

**Keywords:** limited‐resource setting, pediatric surgery, post‐operative care, telehealth

## Abstract

**Background:**

Pediatric operative volumes in Malawi have increased over the past decade due to an increase in the pediatric surgical workforce and improved infrastructure. Despite growth in surgical capacity, limited data exist regarding post‐operative outcomes for common surgical diseases in low‐resource settings.

**Methods:**

Guardians of patients < 18 years old undergoing general surgery operations at Kamuzu Central Hospital (KCH) were recruited. Two telephone call attempts 30‐day after the operation were made to administer a follow‐up questionnaire and an Acceptability of Intervention Measure (AIM) score. Guardians were then purposively sampled for semi‐structured interviews to assess participant experiences in the telephone follow‐up protocol. Feasibility of telehealth follow up, defined here as reachability and acceptability, and guardian perceived factors affecting reachability were analyzed.

**Results:**

Of 327 families approached for recruitment, 309 (94.5%) had access to a phone. After accounting for clinical events, 292 were eligible for a 30‐day phone call. The majority of patients were male (70.6%) and the median age was 2 years old (IQR: 0.02–14). Overall, 69.2% (*n* = 202) were reached via phone call. Participants with secondary phones had increased odds of reachability (OR = 2.0, *p* = 0.04) compared to those with only a primary phone. The mean AIM score of the reached group was 18.3 (SD: 2.6). Among the interviewees—phone ownership, cellular network access, phone charge, and community influence were identified as key factors affecting participation in telephone follow‐up.

**Conclusions:**

Post‐operative follow‐up via phone call is feasible in Malawi as evidenced by high patient reachability and acceptability. The results of this pilot study support scaling up implementation of telehealth follow‐up in Malawi and utilizing the resulting post‐operative metrics to guide quality improvement of surgical care.

## Graphical abstract

Pediatric operative volumes in Malawi have increased over the past decade due to an increase in the pediatric surgical workforce and improved infrastructure. Despite growth in surgical capacity, limited data exist regarding post‐operative outcomes for common surgical diseases in low‐resource settings. Post‐operative follow‐up via phone call is feasible in Malawi as evidenced by high patient reachability and acceptability. The results of this pilot study support scaling up implementation of telehealth follow‐up in Malawi and utilizing the resulting post‐operative metrics to guide quality improvement of surgical care.

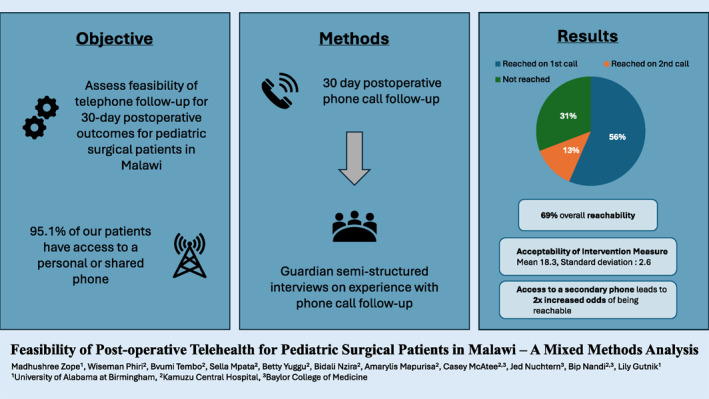

## 1 Introduction

Surgical outcomes monitoring leads to improvement in post‐operative morbidity and mortality by allowing for targeted quality improvement initiatives [1, 2, 3, 4]. In‐hospital perioperative mortality rate is one of six global surgery indicators proposed by the Lancet Commission on Global Surgery (LCoGS) to track progress toward universal access to surgical care by 2030 [5]. Estimates derived from high‐income countries (HICs) attribute approximately 50% of global 30‐day post‐operative mortality to originate from low‐middle income countries (LMICs) despite operative volume being significantly higher in HICs [6]. Many barriers to documenting post‐operative outcomes in LMICs exist such as financing data infrastructure, transport for in‐person clinical evaluations, and workforce burden [7]. Due to these multifaceted barriers, it is often infeasible to follow all post‐operative patients in LMICs, and there is little development of post‐discharge outcomes metrics that may guide improvement in surgical care.

Telehealth, offered in various modalities (e.g., phone calls, video calls, SMS, mobile apps), presents an opportunity to address limitations in monitoring post‐operative outcomes and initiating access to post‐operative care. Malawi, a country of 20.9 million, has an estimated 60 mobile cellular subscriptions per 100 people [8]. Although cell service correlates to areas in proximity of densely populated cities in Malawi, there is increasing service availability in rural areas as well [9]. Utility of telehealth services has been documented in similarly limited‐resource settings (e.g., Rwanda and Haiti) for surgical site infection follow‐up [10, 11].

A national survey of surgical conditions in Malawi reports approximately 26% of children have a surgical condition [12]. Due to an increase in pediatric surgical workforce, specialty operative care is becoming a staple of the health landscape in Malawi [13]. Given the existing scarcity of pediatric surgical outcomes data in Malawi, we conducted a pilot protocol of phone‐call based post‐operative follow‐up at Kamuzu Central Hospital (KCH).

## 2 Methods

### 2.1 Setting

This study took place at the pediatric general surgery department in KCH, a tertiary care public hospital in the capital of Malawi, Lilongwe. It conducts approximately 800 pediatric general surgical operations a year serving a catchment area of ∼12 million children. Each patient has a health passport, a paper book issued by government health facilities, which functions as a longitudinal medical record and is carried by the patient or guardian.

### 2.2 Ethical Considerations

Ethical approval was obtained from the National Health Science and Research Council of Malawi, Institutional Review Board (IRB) of the University of Alabama at Birmingham School of Medicine, and IRB at Baylor College of Medicine. Protected health information was stored in REDCap, and written consent forms were obtained upon enrollment into the telephone follow‐up protocol [14, 15]. A separate written consent form was obtained at the time of the semi‐structured interview administration, and audio recordings of interviews were de‐identified and stored in a password‐protected folder on a computer.

### 2.3 Implementation Outcomes

Using Proctor's Implementation Outcomes Framework, we measured acceptability and feasibility measures within our population [16]. Acceptability at the individual user level was assessed using the validated Acceptability of Intervention Measure (AIM) score [16, 17]. The AIM score is comprised of 4 statements (Telehealth meets my approval, Telehealth is appealing to me, I like Telehealth, I welcome Telehealth), each assigned a Likert scale (1 = completely disagree to 5 = completely agree) of agreeability to the statement. The score ranges from 4 to 20. The AIM tool was translated into Chichewa and verbal responses were recorded. Feasibility at the setting level was assessed using the metric of reachability of guardian via phone call [16].

### 2.4 Telephone Follow Up Protocol and Quantitative Data Collection

Patient guardians were recruited from November 2023–September 2024 at KCH. Eligibility criteria for guardian enrollment included: Patients < 18 years old at the time of operation, access to mobile phones, and fluent in Chichewa, English or both. Reasons for deferral of study participation were documented in the recruitment log. Upon study enrollment, guardians were instructed that they would receive a phone call from our team to evaluate the patient's recovery. Guardians were asked to provide a primary mobile phone number, and if available, a secondary mobile phone number. Secondary phones comprised of any alternative phone line that the guardian had access to through their personal community (relatives, neighbors, etc.), The phone call date was written in each patient's health passport and shown to the patient guardian. The study cell number used by the research assistant to place calls was not provided to the guardian. During the post‐operative hospital stay, demographic, housing and clinical course information was collected. Demographic information to be collected was developed based on questions used for household socioeconomic evaluation in the Demographic and Health Survey [18]. Thirty days after the operation, discharged patients were called by a research assistant with a clinical team member present. If they did not answer, a second phone call was attempted 1 week after. Each attempt involved calling all phone numbers provided at the time of enrollment, once per mobile number. The follow‐up phone call consisted of administering a study specific questionnaire regarding post‐operative symptoms followed by the AIM score (Supporting Information S1: Supplement 1).

### 2.5 Quantitative Data Analysis

Feasibility was defined as patient reachability over 2 phone call attempts per protocol. Using a complete case analysis approach (*n* = 285), multivariable logistic regression was conducted to identify patient characteristics associated with reachability. The English‐speaking‐only patients were excluded from regression modeling due to their small sub‐group size (*n* = 2). A *p*‐value < 0.05 was considered statistically significant. Analyses were conducted using StataSE 18 [19].

### 2.6 Qualitative Data Collection

Purposive sampling of the 30‐day phone follow‐up eligible cohort (*n* = 292) based on reachability, operation type, residence district, sleeping arrangement and water access was conducted to build a list of interviewees. Guardians that responded to and completed a telephone follow‐up call (*n* = 19) and guardians that did not respond to 2 separate call attempts (*n* = 10) were selected to participate in semi‐structured interviews to ensure thematic saturation for interview data. All interviews were conducted in‐person by the research assistant (SM) in Chichewa at KCH in a private office. Participants received the Malawian Kwacha equivalent of 11.50 USD. Written consent was obtained to audio record each participant's interview. A semi‐structured interview guide (Supporting Information S1: Supplement 2) was designed by the surgeons (BN, MZ, LG) and translated into Chichewa (SM). Interviews were audio‐recorded, transcribed verbatim and then translated into English (SM).

### 2.7 Qualitative Data Analysis

The project lead (MZ) conducted an initial round of open coding to develop the codebook using an inductive approach to identify key themes from the transcribed interviews. Transcribed interviews were then coded deductively by two independent reviewers (MZ, SM), using the original open code. The independent reviewers met consistently to maintain continuity and consensus, using a third reviewer (LG) to settle any discrepancies. Using iterative content analysis, prominent themes and quotes were identified from the transcripts. All analyses were completed using Dedoose [19].

## 3 Results

### 3.1 Enrollment in Telephone Follow‐Up Protocol

**FIGURE 1:**
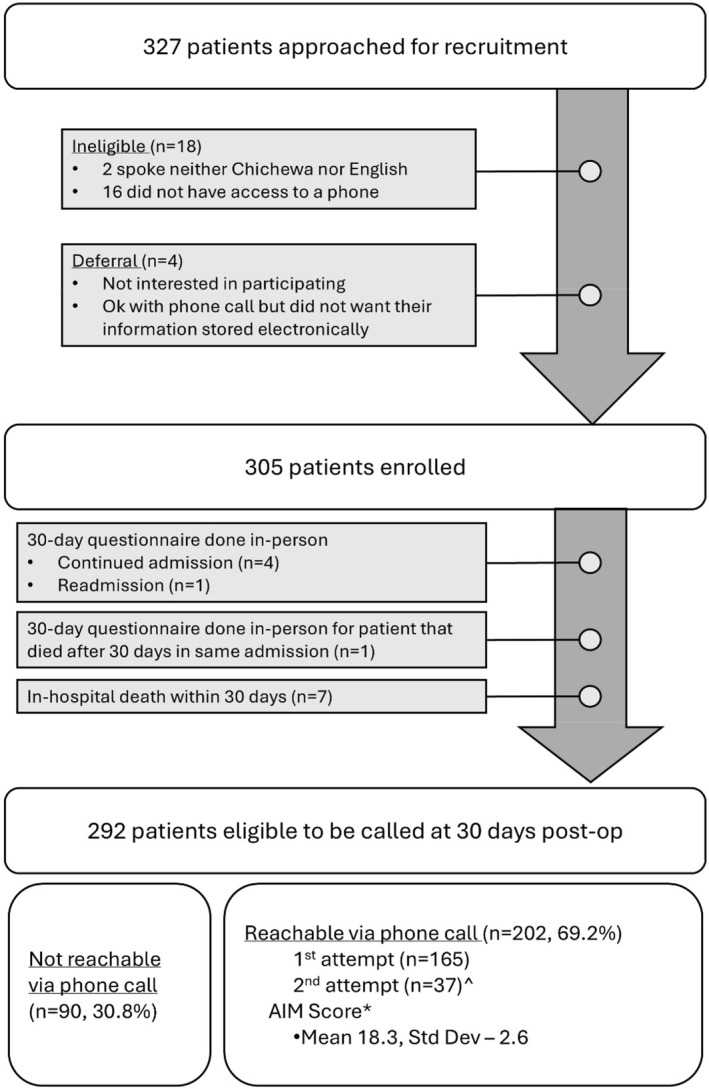
Enrollment and Reachability of study population. The recruitment and enrollment methods are shown. Of the enrolled patients, those eligible for a 30‐day phone call are shown by reachability after 2 separate attempts at calling. ^One patient of this group was reached on second attempt during a second admission. * missing 14.

Three hundred twenty‐seven patients were approached for recruitment. Of these, 16 (4.9%) were ineligible due to not having access to phones, 2 (0.7%) did not speak English and/or Chichewa, and 4 (1.4%) declined study enrollment. Among 305 enrolled, 6 patients participated in an in‐person administration of the 30‐day questionnaire, and 7 patients died during the enrollment admission. Consequently, only 292 patients were eligible to receive a phone call 30 days post‐operatively (Figure 1).

### 3.2 Participant Characteristics

The median patient age of the total patient population (*n* = 292) was 2 years (Table 1A). The median age of patients whose guardians were reached was 3 years old (interquartile range, IQR: 0.02–14) and 1 year old (IQR: 0.01–10) (*p* = 0.03) for those not reached. Overall, 206 (70.6%) patients were male. Most patients were from Malawi (*n* = 290, 99.3%) and of these, 55.2% (*n* = 160) were from the capital district, Lilongwe, where KCH is located. A majority of guardians of patients reported the head of their family sleeping on mattresses (*n* = 127,43.6%) and woven cots (*n* = 92, 45.5%); few reported other arrangements (*n* = 10, 3.4%). A greater proportion of guardians of reached patients reported utilizing mattresses (*n* = 102, 50.5%) versus those not reached (*n* = 25, 28.1%) (*p* < 0.01). Most relied on water access via a borehole, well, or open water source (*n* = 188, 64.6%). However, more guardians of reached patients (*n* = 87, 43.1%) reported having running taps as their daily water source than guardians of not‐reached patients (*n* = 16, 18%) (*p* < 0.001). A majority of guardians of patients were fluent in Chichewa alone (*n* = 240, 82.2%). The reached group had greater proportion of those fluent in Chichewa and English (*n* = 45, 22.3%) than the not reached group (*n* = 5, 5.6%) (< 0.01).

**TABLE 1:** Characteristics of patients by reachability via phone call.

(A) Demographic characteristics of patients that were enrolled for telehealth follow‐up study				
	Total (*n* = 292)	Reached by phone call (*n* = 202)	Not reached by phone call (*n* = 90)	*p*‐value
Age (yrs), median(IQR)^a^				0.03*
By group, *n* (%)	2 (0.01–14)	3 (0.02–14)	2 (0.01–10)	
Neonate (0–30 days)	21 (7.2)	10 (5.0)	11 (12.2)	
Infant (> 30 days to 12 months)	42 (14.3)	24 (11.9)	17 (18.9)	
Toddler (1–6 years)	163 (55.6)	118 (58.4)	45 (50.0)	
Child (> 6 years)	67 (22.9)	50 (22.9)	17 (18.9)	
Sex, *n* (%)				0.69
Female	86 (29.5)	58 (28.7)	28 (31.1)	
Male	206 (70.6)	144 (71.3)	62 (68.9)	
Home residence, *n* (%)				
Country				0.26
Malawi	290 (99.3)	201 (99.5)	89 (98.9)	
Mozambique	1 (0.3)	—	1 (1.1)	
Other	1 (0.3)	1 (0.5)	—	
District				0.11
Lilongwe	160 (55.2)	117 (58.2)	43 (48.3)	
Other	130 (44.8)	84 (41.8)	46 (51.7)	
Housing amenities, *n* (%)				
Sleeping arrangement^a^				< 0.01*
Mattress	127 (43.6)	102 (50.5)	25 (28.1)	
Woven cot	92 (45.5)	92 (45.5)	62 (69.7)	
Other (dirt floor, couch, plastic wrapper,etc)	10 (3.4)	8 (4.0)	2 (2.3)	
Water access^a^				< 0.001*
Running tap	103 (35.4)	87 (43.1)	16 (18.0)	
Other (borehole, well, river/stream/lake, reservoir, community kiosk)	188 (64.6)	115 (56.9)	73 (82.0)	
Patient language, *n* (%)				< 0.01*
Speak chichewa	240 (82.2)	156 (77.2)	84 (93.3)	
Speak English	2 (0.7)	1 (0.5)	1 (1.1)	
Speak both	50 (17.1)	45 (22.3)	5 (5.6)	
Primary phone, *n*(%)				
Owner				< 0.001*
Personal^a^	207 (71.1)	152 (75.6)	55 (61.1)	
Shared^a^	84 (28.9)	49 (24.4)	35 (38.9)	
Type				< 0.001*
Feature^b^	206 (71.0)	132 (65.4)	74 (84.1)	
Smart^b^	84 (29.0)	70 (34.7)	14 (15.9)	
Secondary phone, *n* (%)^c^				< 0.001*
Owner	133 (45.6)	106 (52.5)	27 (30.0)	0.49
Personal	47 (35.3)	39 (36.8)	8 (29.6)	
Shared	86 (64.7)	67 (63.2)	19 (70.4)	
Type				0.01*
Feature^a^	72 (54.6)	52 (49.1)	20 (76.9)	
Smart^a^	60 (45.5)	54 (50.9)	6 (23.1)	

(B) Clinical characteristics of patients that were enrolled for telehealth follow‐up study				
	Total (*n* = 292)	Reached by phone call (*n* = 202)	Not reached by phone call (*n* = 90)	*p*‐value
History of prior operation, *n* (%)	63 (21.6)	37 (18.3)	26 (28.9)	0.04*
Emergent operation, *n* (%)	46 (15.8)	29 (14.4)	17 (18.9)	0.33
Operation type, *n* (%)				0.44
Major	133 (45.6)	89 (44.1)	44 (48.9)	
Hypospadias repair	12 (4.1)	12 (5.9)	—	
Ostomy reversal	22 (7.5)	11 (5.4)	10 (0.11)	
Ostomy formation	7 (2.4)	—	7 (7.8)	
Minor	159 (54.5)	113 (55.9)	46 (51.1)	
Inguinal or umbilical hernia repair	88 (30.1)	68 (33.7)	20 (22.2)	
Orchidopexy	22 (7.5)	15 (7.4)	6 (6.7)	
Rectal biopsy	12 (4.1)	5 (2.5)	7 (7.8)	
Post‐op level of care, *n* (%)				0.94
Low	238 (81.5)	165 (81.7)	73 (81.1)	
Intermediate	11 (3.8)	8 (4.0)	3 (3.3)	
High	43 (14.7)	29 (14.4)	14 (15.6)	
In‐hospital complications, *n* (%)	39 (13.4)	22 (10.9)	17 (18.9)	0.06
In‐hospital outcome, *n* (%)^a^				0.51
Discharge	288 (99.0)	199 (98.5)	89 (100)	
Palliative discharge	1 (0.3)	1 (0.5)	—	
Left AMA	2 (0.7)	2 (1.0)	—	

*Note:* The most common operations for each category (major and minor) are listed. Column % are shown. *p*‐values are calculated by chi‐square testing or mann‐whitney‐u testing.

Abbreviations: AMA = against medical advice; IQR, interquartile range.

^a^ Missing 1.

^b^ Missing 2.

^c^ Column percent for relevant cell total of patients with access to alternate phones shown.

^*^ *p* < 0.05 is denoted as significant.

Nearly all guardians had access to a primary phone (*n* = 311, 95.1%) (Figure 1). Phones were categorized as smartphones or feature phones (i.e., no capability for internet access). A majority of guardians reported their primary phone was personally owned (*n* = 207, 71.1%) and a feature phone (*n* = 132, 65.4%) (Table 1A). The reached group had a higher proportion of personal ownership of the device (75.6%) than the not‐reached group (61.1%, *p* < 0.001). The reached group also had a higher proportion of smartphones (34.7%) than the not‐reached group (15.9%, *p* < 0.001). A total of 133 (45.6%) guardians of enrolled patients had access to a secondary phone. This was more common for the reached group (*n* = 106, 52.5%) than the not reached group (*n* = 27, 30%) (*p* < 0.001). Of those that had access to a secondary phone, 86 (64.7%) reported shared ownership of the device. Reachable guardians had more secondary smartphones (*n* = 54, 50.9%) accessible than non‐reachable guardians (*n* = 6,23.1%) (*p* = 0.01).

Guardians reached by phone had less patients with history of prior operation (*n* = 37,18.3%) compared to their not reached counterparts (*n* = 26,28.9%) (Table 1B). There was a similar distribution of emergent operations and major and minor surgeries among enrollees. Most patients were admitted to a low‐level of care (*n* = 238, 81.5%) and 13.4% overall had complications during their post‐operative hospital admission (*n* = 39). The differences between levels of care are detailed in Supporting Information S1: Supplement 3.

### 3.3 Reachability and Acceptability of Post‐Operative Follow‐Up

**FIGURE 2:**
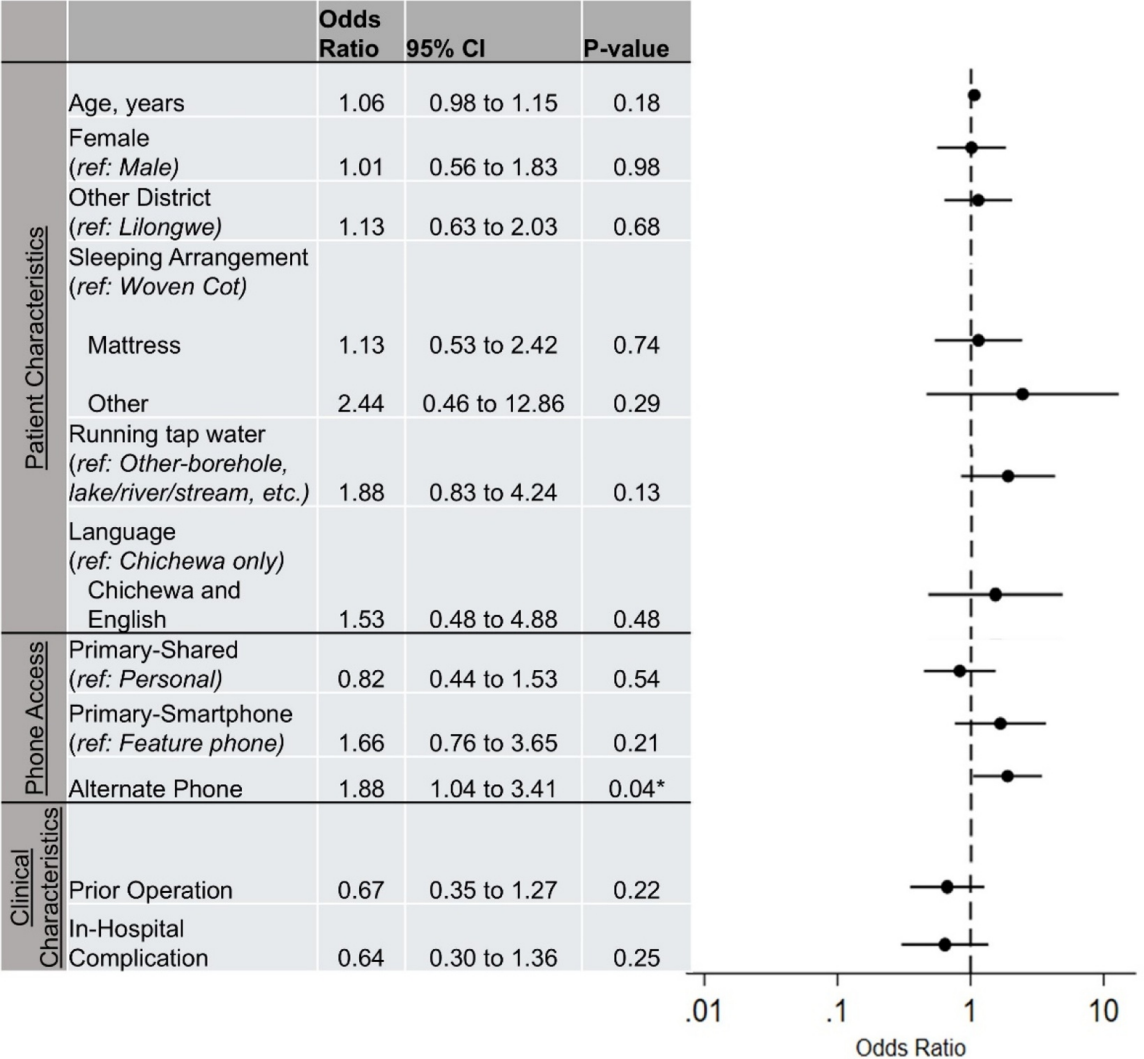
Predictors of telehealth reachability. Multivariable logistic regression analysis of 285 patients' pertinent factors that affect patient reachability via phone call at 30 days post‐op. *p*‐value < 0.05 is denoted as significant*.

There was a cumulative 69.2% (*n* = 202) success rate in telephone follow‐up of the eligible cohort of 292 patients (Figure 1). Of those successfully reached via phone call, 81.7% responded on the first call attempt and 18.3% responded on the second call attempt. Upon multivariable logistic regression, having access to an alternate phone alone was a significant predictor in being reached via phone call (OR = 2.0, *p* = 0.04) (Figure 2). The mean AIM score for the reached group was 18.3 (SD: 2.6) from a possible score range of 4–20, suggesting high acceptability (Figure 1).

### 3.4 Guardian Perceptions of Factors Determining Reachability via Telephone Call

We conducted 29 semi‐structured interviews (19 reachable, 10 not reachable). The characteristics of the interviewee cohort are presented in Table 2. The emerging themes of phone ownership, cellular network access, ability to keep phone charge, and community influence are described with correlating subthemes.

**TABLE 2:** Characteristics of interviewees by reachability via phone call.

(A) Demographic characteristics of interviewees			
	Total (*n* = 29)	Reached by phone call (*n* = 19)	Not reached by phone call (*n* = 10)
Individuals presenting on day of interview, *n* (%)			
Mother alone	5 (17.2)	4 (21.1)	1 (10.0)
Mother, patient, ± sibling of patient	17 (58.6)	10 (52.6)	7 (70.0)
Father alone	4 (13.8)	3 (15.8)	1 (10.0)
Both parents	1 (3.4)	1 (5.3)	—
Both parents, patient	2 (6.9)	1 (5.3)	1 (10.0)
Home residence, *n* (%)			
District in Malawi			
Lilongwe	15 (51.7)	10 (52.6)	5 (50.0)
Other	14 (48.3)	9 (47.4)	5 (50.0)
Housing amenities, *n* (%)^a^			
Sleeping arrangement			
Mattress	11 (37.9)	8 (42.1)	3 (30.0)
Woven cot	18 (62.1)	11 (57.9)	7 (70.0)
Water access			
Running tap	15 (51.7)	6 (31.6)	2 (20.0)
Other (borehole, well, river/stream/lake, reservoir, community kiosk)	14 (48.3)	13 (68.4)	8 (80.0)
Patient language, *n* (%)			
Speak chichewa	25 (86.2)	15 (78.9)	10 (100.0)
Speak both	4 (13.8)	4 (21.1)	—
Primary phone, *n* (%)^a^			
Owner			
Personal	17 (58.6)	14 (73.7)	3 (30.0)
Shared	12 (41.4)	5 (26.3)	7 (70.0)
Type			
Feature	19 (65.6)	10 (52.6)	9 (90.0)
Smart	10 (34.5)	9 (47.4)	1 (10.0)
Secondary phone, *n* (%)	13 (46.4)	9 (47.4)	4 (44.4)
Owner			
Personal	5 (38.5)	4 (44.4)	1 (25.0)
Shared	8 (61.5)	5 (55.6)	3 (75.0)
Type			
Feature	5 (38.5)	3 (33.3)	2 (50.0)
Smart	8 (61.5)	6 (66.7)	2 (50.0)

(B) Clinical characteristics of interviewees			
	Total (*n* = 29)	Reached by phone call (*n* = 19)	Not reached by phone call (*n* = 10)
History of prior operation, *n* (%)	6 (20.7)	3 (15.8)	3 (30)
Emergent operation, *n* (%)	6 (20.7)	6 (31.6)	—
Operation type, *n* (%)			
Major	13 (44.8)	8 (42.1)	5 (50.0)
Minor	16 (55.2)	11 (57.9)	5 (50.0)

*Note:* Column % are shown.

^a^ Column percent for relevant cell total of patients with access to alternate phones shown.

Phone ownership, cellular network access, ability to keep phone charge and community influence were identified as key factors affecting reachability amongst guardian interviews (Table 3). Guardians noted personal versus shared ownership affected their ability to pick‐up the telephone follow‐up at the time of the call; having to rely on a shared phone required the guardian to instruct the owner of the phone to expect a call from KCH, and subsequently have the owner notify the guardian in‐person. Cellular network access was tenuous for receiving calls. Some guardians noted that even if network was available to receive the call, the audio quality was limited and caused difficulty in participating in the follow‐up questionnaire. Similarly, the ability to keep a phone charged was inconsistent for the interviewed cohort. A few guardians noted unreliable electricity access which in turn limited how often their phone was powered on. Community perceptions leading up to the telephone follow‐up varied from being skeptical of unknown callers and discouraging guardians to participate in calls so as not to get caught up in scams, to being surprised, excited, and supportive of the guardian's involvement in this novel protocol. During the interview, guardians also noted that secondary to inconsistent phone charge and network access, they did not use their phones very often for any type of communication (family, business, internet surfing, etc.).

**TABLE 3:** Factors affecting reachability divided into subthemes that recurred over 29 semi‐structured interviews of guardians.

Factors affecting reachability	Subtheme	Representative guardian quote	
		Reachable	Not reachable
Phone ownership	Personal ownership	*“when you called, you called for the first time, at that time the phone was in the house and I was outside. I was doing laundry, so I didn't hear it. And when I went inside the house, I was checking the time and I found the missed call. That was when I was calling. Sure”* (Mother, ID195)	*“Yes, I indeed give them my phone to use because some say that, “can you lend me your phone? I want to walk to someone. We should ask how they are in the hospital. Stuff like that.” So they put in their number and call them, yeah! but it rarely happens.*” (Mother, ID35)
	Shared ownership	*“Ah! I told my family and one guardian who waited for us here in the hospital, the one who gave you the number was there and I just gave a report, “those people have called. They said that I should speak with them.”* (Father, ID200)	*“As I am saying, the phone is for my in‐law, so her husband has more of an envious life for sure. So it is possible that since it's at some people's place, she was trying it. I verily told her that if they call, that they want [patient's name] mother, I should tell her… you should tell me. At the time they didn't pick up, it could have been that she was further away from me.”* (Mother, ID40)
Cellular network access	Reliable cellular network access	*“No. You didn't call at the wrong time. There was also a network.”* (Mother, ID138)	*“Because from my place, it doesn't work from here and Mozambique. If it was at Dedza”* (a malawian border district)*, “maybe the number would have been working.”* (Mother, ID4_92)
	Unreliable cellular network access	*“Yes, at that time the network was jamming, stuff like that. That is because the location network is poor. Some of the things I was able to hear, and some things I couldn't hear clearly.”* (Mother, ID78)	*“What can be challenging to follow up on the phone, mostly I think it's a problem with the network. Because there are other places where the patient may be in a place for the network to work so that you can connect, one has to climb up, he should be on a hill somewhere. So that can be a barrier to follow‐up the patient.”* (Mother, ID97)
Phone charge	Consistently had access to a charged phone	*“I use it everyday. It works for 7 days. Interviewer: All day? Interviewee: Yes!”* (Mother, ID161)	*“My phone's battery is dead. So I use my cousin's, and it is reliable.”* (Mother, ID110)
	Phone was not always charged	*“sometimes the power in the phone goes out, things that cause frequent disconnections.“* (Mother, ID162)	*“No, that's what I'm saying, sometimes it is off. Sometimes the phone is off and the power is off. So charging at our place, we do not have lights. It is powered by solar.”* (Mother, ID4_92)
Community involvement	Guardian perceptions on informing community about the telephone post operative follow up call	*“I thought that this was confidential that I wasn't able to talk to anyone about. For there are some people telling them a thing, they say, “Eh! Do you know, they are saying that at this place this.. Like here at KCH, ‘they are saying that at KCH, some things have started happening? Eh! Do you know, this has started happening at central.’ Perhaps it was something that we could easily spread when it is confidential. And you disrupt something when it is just okay that some could benefit from and you disrupt it.”* (Mother, ID138)	*“I only explained [the telephone follow‐up call was happening] to my family, but I didn't explain to anyone in my community. But my family knows. but in my community I did not explain. Surely.”* (Father, ID62)
	Distrust in credibility of novel telehealth protocol	*“Ah! The time I received the call, it was unknown. I didn't know who it was. But as I have said that we are business people, we receive any phone call. And by the time I answered your call, I was thinking that I would hear some news. That's when I heard that, “we are hospital staff in Kamuzu Central,” I was happy to hear what you have come up with. It was when I answered the phone that I heard the points you brought, and I see that they are beneficial for our lives. Yeah. “*(Father, ID200)	*“It is possible that my wife didn't hear the phone call. Since most of the time, at that time when she was oftenly coming here at the hospital, it was with her. So, ah! I think it's possible that she saw it as a strange number, “ah! Who is this? Eh! When my husband comes, won't he shout at me?”* (Father, ID35)
	Positive and encouraging	*“they [community members] were also happy that, “Ah! Is there even this method of communicating with doctors?” I tell them yes. Well, with my son, it is good, because the doctors are also happy that their patient is doing well, yes. (Laughs) …Well, they felt very good…Full of excitement. (laughs) They also desired that, Oh! Are you talking to doctors?” and I said, ‘Yes! It is possible to speak with doctors.’”* (Mother, ID183)	*“They [community members] were also unexpected, “ah. They helped us until they gave us a phone call?” They didn't believe it. That, “Ah. They have helped us and even are calling us, instead of us going there? …Yes they were supportive. They knew the child's health, so they were supportive.”* (Father, ID62)

*Note:* Representative quotes from each subtheme are shown. Quotes are separated by reachable and non‐reachable guardians interviewed.

## 4 Discussion

Our pilot telephone follow‐up was designed to address the gap in post‐operative post‐discharge outcomes. Standard practice at the time of the proposed study was to recommend in‐person follow‐up as needed or at a specified time based on operation conducted, where the patient lived, and whether they were going to be back at KCH for other medical services. However, there was no longitudinal, collated information system to measure post‐operative outcomes. Conducting in‐person follow‐up visits was also difficult due to the transport costs for the patients.

Piloting a telephone follow‐up protocol demonstrated 94.5% phone access—either by personal or shared phones—and 71.1% of primary phones were personally owned, a metric consistent with the estimated 60 mobile cell subscriptions per 100 persons in Malawi [8]. We reported 69.2% success in being able to reach patients via phone call 30 days after an operation over 2 discrete call attempts. Success rates around 80%–87% reachability have been reported using a similar protocol in Ethiopia, a country with comparable (53) mobile network subscription per 100 people to our study setting in Malawi [20, 21]. An international telephone follow‐up study specifically looking at surgical site infections at 27–30 days after operation found reachability rates ranging from 59% to 97% in 5 LICs and LMICs across WHO defined regions [22]. In Malawi, other telehealth modalities such as two‐way‐texting in an interactive longitudinal format have been used for HIV care and reported 44% of the studied community being eligible due to literacy and phone access limitations [23]. Subsequent participant response in this study was 32%–39%, depending on the type of text message (motivational, visit reminders, etc.) sent. Telephone reachability is highly variable by context of study population, despite similarities in mobile network subscriptions across nations and similar protocols being implemented across different patient populations within the same country. Variability may in part be attributable to reliable electricity access and functional necessity of telephone usage in daily life across communities.

Within the study population, participants with secondary phones were twice as likely to be able to answer a follow‐up phone call. This finding may provide impetus to standardize collection of alternate phone access metrics, personal or shared, in telehealth methodology. To our knowledge, there are few data correlating patient demographic and clinical characteristics to success of telehealth mediated provider to patient communication; however, presence of standardized training of telehealth providers, context specific design, and involvement of stakeholders prior to implementation are all factors that bolster effectiveness of telehealth interventions in Malawi [23, 24] and other LMICs [21, 25, 26]. Development of digital health interventions based on input from patients and healthcare workers to contextualize messaging, and space communication at appropriate intervals was crucial to developing a 2‐way text program in Malawi [27]. The use of telemedicine in surgical care is not a novel intervention in LMICs. Limited published data shows that telephone calls, video calls, and WhatsApp chats are the most used modalities [28]. Of available literature, 41% originates from the Western Pacific region. There is a comparative dearth of information on surgical telehealth in Sub‐Saharan Africa (excluding South Africa). Despite being able to provide some degree of clinical reach into the community, telehealth remains a difficult to adopt option for many LMIC due to barriers in implementation and sustainability.

Guardians identified phone ownership, cellular network access, ability to keep phone charge, and community perceptions to be key factors explaining patterns of reachability. The role of network access and electricity to supply phone charge are well corroborated in implementation of telehealth in low‐resource settings globally [29, 30]. To our knowledge, limited data exist regarding the relative relationships between one phone number, how many individuals rely on it, how geographically dispersed these individuals are, and what the relationships between the primary owner and the individuals are. Phone access in the context of ownership is culturally contextual and complex. Our study population has phone access via personal and shared cellular devices which may support telephone follow‐up implementation due to having more individual persons connected to a phone line population wide; however, it can be difficult for the owner of a shared phone to contact the guardian of the child if they are not in immediate vicinity at the time of the call.

Some qualitative studies on maternal‐child health in Malawi have corroborated the integral role of family and community in a patient's health beliefs. For example, the societal authority held by elder women in communities compels mothers to choose birthing methods based on their elders' advice [31, 32]. Family and community perceptions have also been shown to dictate patient's decision to seek medical care in Ghana and Uganda [33, 34]. Community members in Uganda state that knowing neighbors who have undergone successful operations makes the entire community more amenable to considering consenting to operative treatment. The role of community perceptions on influencing a patient's health beliefs and behaviors has been recognized a key factor in supporting novel intervention uptake within the global health setting [34, 35].

There are limited data on assessing phone usage patterns (how often, what for, what type, etc.) in Malawi. Our work adds to these data by documenting frequency of phone usage in the study population. These nuances of phone ownership suggest that targeted studies on shared phone numbers and community involvement in post‐operative care may be necessary to understand how effective telephone follow‐up may be in a given cultural context.

This study has several limitations. Clinical evaluation of patients with concerning post‐operative symptoms was limited to verbal communication alone. Visual methods, such as video calls or picture sharing, were not undertaken. Phone call attempts were also limited to 1 call per provided telephone number 30 days after the operation, followed by a repeat attempt using the same method 7 days later. This protocol was chosen taking into consideration human resource constraints should the method prove feasible and garner stakeholder interest in continuing the practice. Increasing the number of phone call attempts may have increased the number of patients reached in the study population. However, it is important to note that the involvement of a clinician was crucial during phone calls made by the research team to aid in triaging any clinical concerns that required recommending in‐person evaluation at a nearby health facility. Future continued use will require optimizing this workflow to function within the time constraints of the existing clinical team within the department. Success rates in reachability for post‐operative follow‐up are likely highly dependent on access to cellular network and electricity to maintain phone charge. This limits the permeability of using phone call follow‐up practices in settings where network and electricity are persistently unavailable.

## 5 Conclusion

Post‐operative tele‐health follow up via phone call is feasible in Malawi as evidenced by high reachability and acceptability. Phone ownership, cellular network access, capability of keeping phone charge, and community influence were identified by guardians as key factors in reachability. The results of this pilot study provide impetus to scale up implementation of telehealth follow‐up and utilize the resulting post‐operative metrics to guide quality improvement of surgical care.

## Author Contributions

**Madhushree Zope:** conceptualization, methodology, data curation, software, investigation, validation, formal analysis, funding acquisition, project administration, writing – original draft, writing – review and editing, resources, visualization. **Seraiah Mdala:** methodology, investigation, validation, formal analysis, writing – review and editing, project administration, resources. **Wiseman Phiri:** methodology, validation, data curation, project administration, writing – review and editing. **Bvumi Tembo:** methodology, data curation, validation, project administration, writing – review and editing. **Sella Mpata:** methodology, validation, project administration, writing – review and editing, data curation. **Betty Yuggu:** methodology, data curation, validation, project administration, writing – review and editing. **Bidali Nzira:** methodology, data curation, validation, project administration, writing – review and editing. **Amarylis Mapurisa:** conceptualization, methodology, data curation, validation, project administration, supervision, writing – review and editing. **Casey McAtee L:** conceptualization, methodology, supervision, writing – review and editing, funding acquisition. **Jed G: Nuchtern:** conceptualization, funding acquisition, writing – review and editing, methodology, supervision. **Bip Nandi:** conceptualization, methodology, validation, data curation, supervision, funding acquisition, project administration, resources, writing – review and editing. **Lily Gutnik:** conceptualization, methodology, data curation, validation, supervision, funding acquisition, project administration, resources, writing – review and editing.

## Funding

Fogarty International Center of the National Institutes of Health (NIH), Award Number D43TW012274.

## Consent

Informed consent was obtained from all individual participants included in the study.

## Conflicts of Interest

The authors declare no conflicts of interest.

## Supporting information


Supporting Information S1

## Data Availability

Research data are not shared.
