# Supplementary material for: Feasibility of Post‐Operative Telehealth for Pediatric Surgical Patients in Malawi—A Mixed Methods Analysis
Source: World J Surg. 2026 Jun 30;50(8):2360–71. doi: 10.1002/wjs.70448 (PMC13460689; doi:10.1002/wjs.70448)
Supplement: Supplementary file 1 [file WJS-50-2360-s001.docx]

**Supplement 1: 30-day follow-up questionnaire and AIM Score**

| **Procedure** | **Assessment goal** | **Two week follow up question** |
| --- | --- | --- |
| All procedures | 1. Patient perception of postoperative recovery 2. Is the patient alive | 1. How is your child doing since the surgery?  2. Is anything bothering them?  3. Has their activity returned to as it was before surgery (i.e., able to walk, play, attend school, do chores)? |
| All procedures | 1. Postoperative medical visits  2. Surgical site infection  3. Pain control  4. Postoperative fever | 1. Did you want to see a medical professional in the last 30 days about your child’s health?    a. If yes, did you go to see someone?    b. If yes, who did you see?    c. If yes, what treatment did they give you? 2. Do you have concerns about your child’s incision? 3. Have you noticed redness at the incision? 4. Is there pus at the incision? 5. Is there swelling around the surgical incision? 6. Did your child have pain after discharge? 7. Has your child had any fevers, chills, night sweats that have concerned you since the surgery? 8. If yes to any one of 1-7    a. how many days has this been happening?    b. Is it getting better or worse? |
| Umbilical hernia repair, Inguinal hernia repair specific | 1. Hernia recurrence | 1. Has the bulge at the belly button or groin returned? 2. If yes to 1    a. how many days has this been happening?    b. Is it getting better or worse? |
| Gastrointestinal surgery (appendectomy, perforated typhus, etc.) | 1. Bowel function 2. Oral intake | 1. Is your child having regular bowel movements? 2. Does your child have a stoma?    a. If yes, is the stoma sticking out more than usual?    b. If yes, is the stoma sinking into the skin?    c. If yes, is the stoma bleeding?    d. If yes, have you noticed any problems in the skin around the stoma? 3. Is your child having loose stools?    a. If yes, how many times a day? 4. Is your child constipated?    a. If yes, how many days ago was the last stool? 5. Is your child’s appetite back to normal? 6. Has your child been vomiting?    a. If yes, What color?    b. If yes, how often? Times/week 7. Is your child’s abdomen swollen? 8. Have you noticed weight loss in your child? |
| Urologic surgery (hydrocele repair, orchiopexy, hypospadias, PUJ obstruction, PUV etc.) | 1. Swelling at operative site 2. Urinary habits | 1. Is your child having problems with urination? 2. Is your child urinating daily? 3. Do they have pain when urinating? 4. Do they have clear yellow urine? 5. Do they have pus or white colored urine? 6. Do they have red or pink colored urine? 7. Have you noticed worsening swelling of the scrotum or penis? 8. Have you noticed redness at the scrotum or penis? |

I will now ask you to rate your experience with the telehealth follow-up program. Please tell me on a scale of 1 to 5 whether you disagree or agree with the statements. 1 means you completely disagree, 2 means you disagree, 3 means you neither agree nor disagree, 4 means you agree, 5 means you completely agree.

The first statement is that Telehealth follow-up meets my approval. How would you rate this on a scale of 1 to 5?

The second statement is that Telehealth follow-up is appealing to me. How would you rate this on a scale of 1 to 5?

The third statement is that I like Telehealth follow-up. How would you rate this on a scale of 1 to 5?

The last statement is that I welcome Telehealth follow-up. How would you rate this on a scale of 1 to 5?

**Supplement 2: Semi-structured interview guide** for reachable and non-reachable guardians in the telephone follow-up protocol. Questions only asked to reachable guardians are underlined. Questions only asked to non-reachable guardians are *italicized.*

Hello, I am [insert name]. Thank you for agreeing to talk with me today. I am part of the pediatric surgery department at Kamuzu Central Hospital. We are working on creating a telehealth follow-up protocol for pediatric surgical patients. The goal of this research is to understand how patients feel about follow-up after surgery and get their ideas on ways to conduct the follow-up. It is very important for us to hear your opinions.

Whether or not you would like to participate in this in person interview is completely up to you. You don’t have to answer questions you don’t want to. This conversation will be recorded so that we do not miss anything you say. Your opinion will be kept confidential and will not affect the medical care that you receive. Your name will not be used in our research and no one will be able to identify your answers.

Do I have your permission to record this discussion?

*Start recorder*

Hello again, my name is [insert name]. I work with the pediatric surgery department at Kamuzu Central Hospital. Thank you for agreeing to participate in our interview for this study. Do you have any questions before we start?

1. Tell me about your experience when your child had surgery, particularly after the surgery was over. How did you feel?

- Probe:
- Did you interact much with the doctors or nurses when your child was in the hospital?
- How were those interactions?
- What was good and what was bad?

2. Was there a plan for follow-up visits scheduled after the operation?
   - Probe: Did you agree with this plan? Did you think that you would be able to be present for the follow-up?
   - how did you plan to attend in-person follow-up?
   - who did you call to arrange for transport?
   - did you have to take time off from work?
   - did the child (if old enough) have to take time off from school or home errands?
3. Did your child have any problems after the operation? How did you sort out the problem?
4. What are your thoughts on telehealth? This is where someone from the hospital calls you over the phone to see how your child is doing. It is like an in person clinic visit with a health care provider, but limited to the phone.
   - Probe:
   - Please explain your opinions.
   - What are your thoughts on being comfortable talking about your child’s health over phone?
   - What concerns do you have?
   - What are some potential good things about such an approach?
   - Is this something you would want to have as an option for talking to the health care provider?
5. How often do you use your phone and what for?

- Probe:
  - Do you use it to text? How do you text? How often? Multiple times every day? Or just a few times per week?
  - Do you share it with your community? Why? Do community members use your phone number to receive and make calls? Please explain why or why not

6. *How did you prepare for the post-operative phone call?*
   - *Probe:*
   - *Did you know it was going to happen?*
   - *Did you know it would happen in 30 days after surgery?*
   - *Did you know that we tried calling the given number twice?*
   - *Why do you think you were unable to pick the call? Do you have your phone charged for most of the day? Are you able to get network at your place of residence or work?*
7. How was your experience with receiving the follow-up phone call from our team? This call happened a month after your child’s surgery. What did you like? What did you not like?
   - Probe:
   - Was there anything that made it difficult to be able to take the call? (for example: missed call, cell service issues, bad time for the call) if yes, how did you handle the situation?
   - Was this the first time you had a telehealth experience? how did it make you feel?
8. Did you tell your family or community that the call was happening? What did your community and family members think about having a telehealth follow-up after your child’s surgery?
   - Probe:
   - Were they supportive? Why?
   - Were they nervous? Why?
   - Did different people have different reactions?
   - How did other people’s thoughts about this call make you feel?
9. How did you feel after the call?

- Probe:
  - Was it helpful? if yes, in what ways?

10. If you didn’t get the telehealth phone call, would you have wanted for your child to be seen by health care provider? Why or why not?
    - Probe:
    - what symptoms would have made you seek out care for your child? For example, if your child was having fevers and night sweats, would that concern you enough to seek care?
    - What other specific health changes would worry you after an operation for your child?
11. If you thought your child needed to be seen by a health care provider, how would you seek out this care? Would you go back to KCH or go somewhere else?
    - Probe:
    - How would you get transport there?
    - How long would it take you to get there?
    - How would you pay?
    - Would you prefer to come to KCH for this issue?
12. Are there other ways that we can help you make sure that your child is ok after surgery?
    - Probe:
      - For example, mandatory schedule follow up visit?
      - What examples or ideas would you suggest, if any?
13. What are your thoughts about telehealth follow-up being offered for all surgery patients after the operation? What are some good things about this? What are some challenges?
14. Do you have a phone that can download apps (i.e. a smartphone)? Or do you have a regular phone that cannot download apps? What do you think about doing telehealth over text or a mobile app instead of a phone call?

- Probe:
  - If parent has a regular phone: What would telehealth over the phone look like? What options would you like to see over text?
  - If parent has a smartphone: What would you like to see in the app? (i.e. what information?) How would you use the app?

15. Is there anything else you’d like to discuss that we didn’t?

Thank you for participating in our research study. I want to quickly reflect on the topics we covered. We have talked about what the process of getting an operation was like for your child and what your experience was with the telehealth follow-up call. We also discussed the views of families on follow-up after an operation and whether telehealth is a good way to achieve this.

Great, thank you so much for your help and time. Your experience is very valuable and will help our team guide surgical care for children at Kamuzu Central Hospital.

**Supplement 3:** Description of inpatient admission wards and their associated levels of care
